# Resting Energy Expenditure, Insulin Resistance and *UCP1* Expression in Human Subcutaneous and Visceral Adipose Tissue of Patients With Obesity

**DOI:** 10.3389/fendo.2019.00548

**Published:** 2019-08-07

**Authors:** Silvia Bettini, Francesca Favaretto, Chiara Compagnin, Anna Belligoli, Marta Sanna, Roberto Fabris, Roberto Serra, Chiara Dal Prà, Luca Prevedello, Mirto Foletto, Roberto Vettor, Gabriella Milan, Luca Busetto

**Affiliations:** ^1^Internal Medicine 3, Department of Medicine, DIMED, University of Padua, Padua, Italy; ^2^Center for the Study and the Integrated Treatment of Obesity, University Hospital of Padua, Padua, Italy

**Keywords:** resting energy expenditure, obesity, insulin resistance, UCP1, browning, adipose tissue

## Abstract

Determinants of resting energy expenditure (REE) in humans are still under investigation, especially the association with insulin resistance. Brown adipose tissue (AT) regulates energy expenditure through the activity of the uncoupling protein 1 (UCP1). White AT browning is the process by which some adipocytes within AT depots acquire properties of brown adipocytes (“brite” adipocytes) and it correlates with metabolic improvement. We analyzed determinants of REE in patients with obesity and assessed *UCP1* expression as a “brite” marker in abdominal subcutaneous AT (SAT) and visceral omental AT (VAT). Clinical data, REE, free fat mass (FFM), and fat mass (FM) were determined in 209 patients with obesity. *UCP1*, PPARG coactivator 1 alpha (*PPARGC1A*), transcription factor A, mitochondrial (*TFAM*), T-box transcription factor 1 (*TBX1*), and solute carrier family 27 member 1 (*SLC27A1*) expression was assayed in SAT and VAT samples, obtained during sleeve gastrectomy from 62 patients with obesity. REE and body composition data were also available for a subgroup of 35 of whom. In 209 patients with obesity a multiple regression model was computed with REE as the dependent variable and sex, waist, FFM, FM, homeostasis model assessment-insulin resistance (HOMA), interleukin-6 and High Density Lipoprotein-cholesterol as the independent variables. Only FFM, FM and HOMA were independently correlated with REE (*r* = 0.787, AdjRsqr = 0.602). In each patient VAT displayed a higher *UCP1, PPARGC1A, TFAM, TBX1*, and *SLC27A1* expression than SAT and *UCP1* expression in VAT (*UCP1*-VAT) correlated with Body Mass Index (BMI) (*r* = 0.287, *p* < 0.05). Introducing *UCP1*-VAT in the multivariate model, we showed that FFM, HOMA, interleukin-6, High Density Lipoprotein-cholesterol, and *UCP1*-VAT were independent factors correlated with REE (*r* = 0.736, AdjRsqr = 0.612). We confirmed that REE correlates with FFM, FM and HOMA in a large cohort of patients. Our results clearly showed that *UCP1*-VAT expression was significantly increased in severe human obesity (BMI > 50 kg/m^2^) and that it behaved as an independent predictor of REE. Lastly, we suggest that an increased REE and browning in metabolically complicated severe obesity could represent an effort to counteract further weight gain.

## Introduction

Determinants of resting energy expenditure (REE) in humans are still under investigation, especially in patients with severe obesity. Fat free mass (FFM) explains more than 80% of the inter-individual variance in REE (1), but other factors could play a role, such as heritability, hypertension and insulin resistance (2). While an association between REE and insulin resistance (IR) has been previously shown (3–5), in particular in Pima Indians (6), the underlying mechanisms remain unclear.

Brown adipose tissue (BAT) regulates energy expenditure by the dissipation of energy as heat through the activity of the uncoupling protein 1 (UCP1) (7). Several studies have suggested that BAT activation is associated with reduction in blood glucose levels, improvement of IR and increased REE both in animals and in humans (8–10). On the contrary, within white adipose tissue (WAT) depots, visceral adipose tissue (VAT) is a more pathogenic depot compared to subcutaneous adipose tissue (SAT) and increased VAT correlates with a high risk of metabolic syndrome and type 2 diabetes mellitus (T2DM) (11).

**Figure 1:**
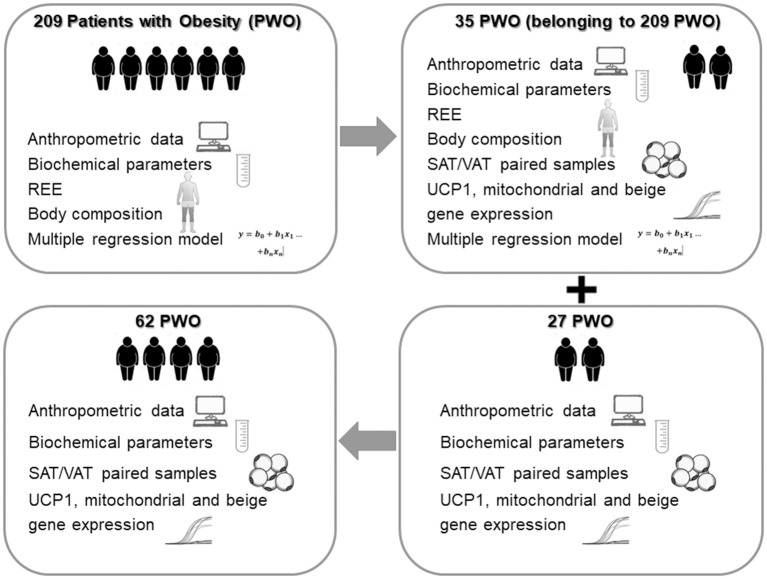
Study design. PWO, Patients with Obesity; REE, resting energy expenditure; SAT, subcutaneous Adipose Tissue; VAT, visceral omental Adipose Tissue; UCP1, uncoupling protein 1.

Browning is the process by which some adipocytes within WAT depots acquire properties of brown adipocytes (called “beige” or “brite” adipocytes) and studies in mice show a correlation between browning and metabolic improvement (12). While the expression of browning genes in mice is greater in SAT compared to VAT, an opposite pattern of browning gene expression with VAT having higher expression than SAT was observed in humans (13, 14). Indeed, *UCP1* expression in VAT and SAT (*UCP1*-VAT and *UCP1*-SAT), particularly in patients with severe obesity, are still controversial. In the present study, we analyze different populations of patients with severe obesity (PWO) (Figure 1), with the aim of identifying new determinants of REE including *UCP1* expression in abdominal SAT and VAT.

## Results

### Analyses of REE in 209 Patients With Obesity

Clinical and laboratory evaluations of the 209 PWO are reported in Table 1. Most of the patients were affected by severe obesity (30 kg/m^2^ ≤ Body Mass Index (BMI) < 34.99 kg/m^2^: *n* = 7, 3.3%; 35 kg/m^2^ ≤ BMI < 39.99 kg/m^2^: *n* = 47, 22.5%; BMI ≥ 40 kg/m^2^: *n* = 155, 74.2%).

**Table 1:** Anthropometric characteristics, biochemical parameters, gene expression analyses in white adipose tissue depots, body composition and resting energy expenditure (REE) in 209 patients with obesity (PWO).

	**209 PWO**	**62 PWO**	**35 PWO**	***p***
Sex (M/F)	95/114	16/46	10/25	ns
Age (y)	45 ± 12	46 ± 12	45 ± 14	ns
Weight (kg)	127.7 ± 24.8	129 ± 21.6	120.5 ± 18.5	ns
BMI (kg/m^2^)	43.9 (39.9–49.4)	45.4 (42.1–53.4)	45.1 ± 8.3	ns
WC (cm)	131 ± 15	132 ± 12	128 ± 12	ns
FPG (mmol/l)	5.8 ± 1.5	5.8 (5.1–7)	5.9 ± 1.5	ns
Insulin (mU/l)	19.9 ± 12.6	19 (13–29)	21.4 ± 13	ns
HOMA	5.27 ± 2.9	4.98 (3.26–7.92)	5.73 ± 2.3	ns
TC (mg/dl)	192 ± 37	189 ± 34	192 ± 34	ns
HDL (mg/dl)	46 ± 12	47 ± 12	48 ± 13	ns
NHDLC (mg/dl)	145 ± 38	142 ± 33	145 ± 38	ns
LDL (mg/dl)	121 (99–138)	119 (95–136)	117 (97–136)	ns
TG (mg/dl)	110 (79–153)	121 (90–167)	126 (89–160)	ns
hsCRP (mg/l)	5.58 (3.15–10.07)	6.6 (3.6–10)	7.2 (3.5–11)	ns
TNF-a (ng/l)	8.2 (6.8–10.6)	8.1 (6.5–9.5)	8 (6.2–9.2)	ns
IL-6 (ng/l)	2.2 (1.9–3.7)	3 (2.1–4.4)	2.9 (2–3.9)	ns
Leptin (μg/l)	34 ± 17	39 ± 16	36.6 ± 15	ns
REE (Kcal/day)	1,964 ± 467	–	1,785 ± 300	ns
RQ	0.79 ± 0.12	–	0.72 ± 0.08	ns
FM (kg)	53.4 ± 14.32	–	54.6 ± 11.9	ns
FM (%)	42.7 ± 8	–	45.6 ± 7	ns
FFM (kg)	71.7 ± 17.1	–	64.6 ± 12.6	ns
FFM (%)	57.4 ± 8.6	–	54.4 ± 7	ns
*UCP1*-SAT	–	0 (0–0.001)	0 (0–0.001)	–
*UCP1*-VAT	–	0.006 (0.004–0.011)	0.008 ± 0.005	–
*PPARGC1A*-SAT	–	1.56 (1.12–2.42)	1.85 (1.3–2.49)	–
*PPARGC1A*-VAT	–	4.5 (2.6–6.6)	3.9 (1.98–6.54)	–
*TFAM*-SAT	–	0.13 (0.09–0.22)	0.14 (0.1–0.23)	–
*TFAM*-VATAT	–	0.35 (0.25–0.52)	0.32 (0.2–0.43)	–
*TBX1*-SAT	–	0.1 (0.06–0.16)	0.09 (0.06–0.14)	–
*TBX1*-VAT	–	0.15 (0.074–0.29)	0.15 (0.07–0.29)	–
*SLC27A1*-SAT	–	0.37 (0.27–0.51)	0.3 (0.27–0.5)	–
*SLC27A1*-VAT	–	0.5 (0.34–0.72)	0.52 ± 0.29	–

*M, male; F, female; BMI, Body Mass Index; WC, waist circumference; FPG, Fasting plasma glucose; HOMA, Homeostasis model assessment-insulin resistance index; TC, total cholesterol; HDL, High Density Lipoprotein –cholesterol; NHDLC, non-HDL-cholesterol; LDL, Low Density Lipoprotein –cholesterol; TG, triglycerides; hsCRP, high-sensitivity C-Reactive Protein; TNF-α, Tumor Necrosis Factor-α; IL-6, interleukin-6; RQ, respiratory quotient; FM, Fat mass; FFM, Free Fat Mass; UCP1, uncoupling protein 1; PPARGC1A, PPARG coactivator 1 alpha; TFAM, transcription factor A, mitochondrial; TBX1, T-box transcription factor 1; SLC27A1, solute carrier family 27 member 1; SAT, subcutaneous adipose tissue; VAT, visceral omental adipose tissue. Data are presented as mean values ± standard deviations when Normality Test (Shapiro-Wilk) and Equal Variance Test (Brown-Forsythe) have been passed or, if not, as median values (25th-75th percentile). Thirty five PWO's data were compared with 209 PWO's data; statistical analysis was performed with the Mann-Whitney test for independent samples in non-normally distributed variables, independent samples t-test in normally distributed variables and Fisher's exact test in categorical variables; results were reported in the p column*.

Self-reported spontaneous physical activity levels were similar among patients.

We divided our population according to their glycaemic profile [based on American Diabetes Association (ADA) (15)] in 3 groups sex and BMI matched: 100 PWO were normoglycaemic, 60 had prediabetes (impaired fasting glycaemia and/or impaired glucose tolerance at the oral glucose tolerance test, OGTT), 49 had T2DM. In the 3 groups REE levels did not display any significant difference even if we found a trend of increase from patients with normoglycaemia [REE = 1,826 (1,618–2,207) kcal/day] to patients with prediabetes [REE = 1,897 (1,498–2,314) kcal/day] and T2DM [REE = 2,015 (1,707–2,340) kcal/day], *p* = 0.404.

The REE values were significantly correlated with: sex (*M* = 1 and *F* = 2; *r* = – 0.591, *p* < 0.001), weight (*r* = 0.731, *p* < 0.001), BMI (*r* = 0.5, *p* < 0.001), waist circumference (WC) (*r* = 0.616, *p* < 0.001), FFM (*r* = 0.73, *p* < 0.001), fat mass (FM) (*r* = 0.294, *p* < 0.001), fasting plasma glucose (FPG) (*r* = 0.203, *p* < 0.01), fasting insulin (*r* = 0.218, *p* < 0.01), homeostasis model assessment-insulin resistance (HOMA) (*r* = 0.239, *p* < 0.001), high-sensitivity C-reactive protein (hs-CRP) (*r* = 0.228, *p* < 0.01), interleukin-6 (IL-6) (*r* = 0.201, *p* < 0.01), and High Density Lipoprotein-cholesterol (HDL-cholesterol) (*r* = −0.242, *p* < 0.001). To investigate the role of different parameters as predictors of REE, a multiple regression model was computed with REE as the dependent variable and sex, WC, FFM, FM, HOMA, IL-6, and HDL-cholesterol as the independent variables. We excluded some interrelated variables: BMI vs. FFM and FM and we selected IL-6 values to include inflammatory markers and HOMA data as cumulative valuable index of IR. In this model, FFM, FM and HOMA were the only variables independently correlated with REE (*r* = 0.785, AdjRsqr = 0.601) (Table 2).

**Table 2:** Correlation and regression analyses of Resting Energy Expenditure (REE) in 209 patients with obesity.

	**REE (kcal/day)**	
	**Simple linear coefficients (*r*)**	**Multiple regression coefficients (β)**
Sex	−0.591^***^	−0.131
WC	0.616^***^	0.0773
FFM	0.73^***^	0.55^***^
FM	0.294^***^	0.199^**^
HOMA	0.239^***^	0.120^*^
HDL	−0.242^***^	0.0598
IL-6	0.201**	0.0785

*WC, waist circumference; FFM, Free Fat Mass (kg); FM, Fat mass (kg); HOMA, Homeostasis model assessment-insulin resistance index; HDL, High Density Lipoprotein –cholesterol; IL-6, interleukin-6. Simple linear correlations were calculated by Pearson's correlation. FM, FFM, and HOMA were independently related to REE in a multiple regression model (r = 0.785, AdjRsqr = 0.601)*.

* p < 0.05;

** p < 0.01;

*** *p < 0.001*.

### Gene Expression Analysis in Adipose Tissue Depots of 62 Patients With Obesity

We quantified mRNA expression of genes related to mitochondria and brown/beige adipocytes in paired SAT and VAT of 62 PWO collected during bariatric surgery procedures. Clinical and laboratory evaluations of this group were reported in Table 1.

**Figure 2:**
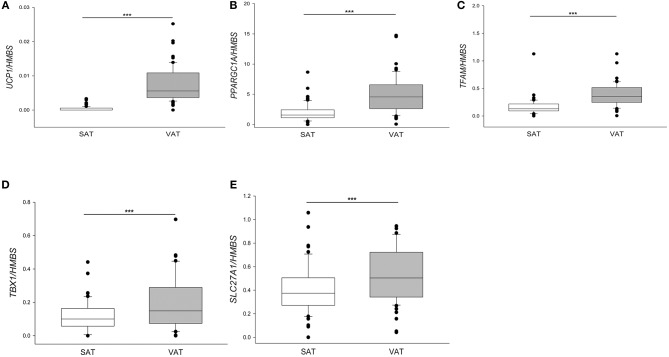
Gene expression in paired subcutaneous and visceral adipose tissue of patients with severe obesity. *UCP1* **(A)**, *PPARGC1A* **(B)**, *TFAM* **(C)**, *TBX1* **(D)**, *SLC27A1* **(E)** mRNA were quantified by qPCR and normalized for *HMBS* mRNA in SAT and VAT depots for each of 62 patients with severe obesity described in Table 1. For *UCP1* expression, the positive samples were 23/62 for SAT and 61/62 for VAT. Results were presented as a box plot, with 25th, 75th percentile and median values. Statistical analysis was performed by the Mann-Whitney *U*-test (***p < 0.0001).

These patients compared to those described in the previous paragraph displayed a higher BMI range [BMI in 62 patients: 45.4 (42.1–53.4) vs. BMI in 209 patients: 43.9 (39.9–49.4), *p* < 0.01], including some patients with very high BMI values, and consequently a higher level of circulating leptin. We assessed that WAT of PWO in basal conditions expressed very low levels of *UCP1* mRNA (10^4^/10^5^ times lower) when compared to human VAT surrounding pheochromocytoma that we used as a positive control. In fact, pheochromocytoma is an adrenal neoplasm secreting high levels of catecholamines which strongly stimulate browning and UCP1 expression in the adipose tissue (16). However, we quantified a higher *UCP1* expression in VAT compared to SAT of the same patient (Figure 2A) and in several SAT biopsies *UCP1* expression resulted undetectable (39/62). In agreement with high *UCP1* expression, VAT expressed also higher levels of *PPARGC1A* and *TFAM* than SAT (Figures 2B,C). Visceral tissue displayed also a significant increase of the beige-related markers *TBX1* and *SLC27A1* (Figures 2D,E) when compared to paired SAT.

**Figure 3:**
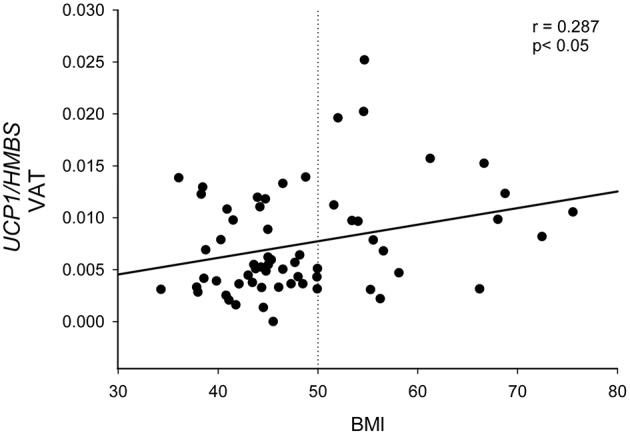
Correlation between *UCP1* expression in visceral omental adipose tissue and BMI. *UCP1* mRNA expression quantified by qPCR and normalized to *HMBS* mRNA was correlated with BMI in 61 patients with severe obesity. Statistical analysis was performed by Pearson correlation. The dotted line represents the BMI cut-off of 50 kg/m^2^ used to divide patients in Figure 4.

*UCP1*-VAT correlated with BMI (*r* = 0.287, *p* < 0.05), as shown in Figure 3.

No further simple correlations were found between *UCP1*-VAT or *UCP1*-SAT and other clinical and laboratory parameters reported in Table 1. It is worth noting that we highlighted a strong correlations between VAT expression of *UCP1* and *PPARGC1A* (*r* = 0.465, *p* < 0.001), *TFAM* (*r* = 0.455, *p* < 0.001), *TBX1* (*r* = 0.426, *p* < 0.001), and *SLC27A1* (*r* = 0.374, *p* < 0.01).

**Figure 4:**
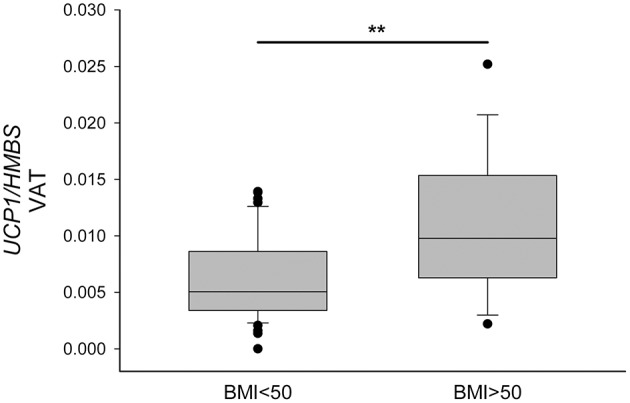
Increased UCP1 expression in visceral omental adipose tissue of patients with BMI higher than 50 kg/m^2^. *UCP1* mRNA was quantified in VAT by qPCR, normalized for HMBS and compared in PWO with BMI lower than 50 (*n* = 43) and with BMI higher that 50 (*n* = 18). Data were reported as a box plot, with 25th, 75th percentile and median values. Statistical analysis was performed by the Mann-Whitney *U*-test (**p < 0.01).

On the basis on the distribution of the *UCP1*-VAT showed in Figure 3, we divided the PWO into two groups using the BMI cut-off of 50 kg/m^2^. In this way, we highlighted that PWO with BMI higher than 50 (*n* = 18) were characterized by a significantly higher expression of *UCP1* in VAT depot compared to PWO with BMI lower than 50 (*n* = 43) in basal conditions (*p* = 0.007) (Figure 4).

### *UCP1* Expression in Visceral Omental Adipose Tissue As a Predictor of REE

In order to study any correlation between *UCP1*-VAT and REE, we analyzed a subgroup of PWO (*n* = 35), belonging to the main population of 209 PWO where both *UCP1* expression and indirect calorimetry for REE quantification were available (Figure 1). The clinical and laboratory parameters of these patients were described in Table 1 and compared with patients of the main population. The statistical analysis clearly showed that the anthropometric characteristics, the biochemical parameters, the body composition and the REE of this subgroup of 35 PWO did not statistically differ from the main population of 209 PWO (p column of Table 1).

In this subgroup of 35 PWO, we confirmed the correlations between REE and weight (*r* = 0.497, *p* < 0.01), BMI (*r* = 0.366, *p* < 0.05), WC (*r* = 0.406, *p* < 0.05), FFM (*r* = 0.45, *p* < 0.01) and HOMA (*r* = 0.472, *p* < 0.01). Thus, we applied to this subgroup the same multiple regression model computed for the main population of 209 patients introducing in the analysis the new *UCP1*-VAT variable as a possible biological implicating factor. *PPARGC1A, TFAM, TBX1* and *SLC27A1* expression values in VAT were excluded from this model because of their multicollinearity each other and with *UCP*-VAT. In this new analysis FFM, HOMA, IL-6, HDL-cholesterol and *UCP1*-VAT were the five independent factors correlated with REE (*r* = 0.736, AdjRsqr = 0.612) (Table 3). Thus, we confirmed the role of FFM and HOMA, previously obtained analyzing the main population of 209 PWO, but we also highlighted a possible independent contribution of *UCP1*-VAT as a determinant of REE. In particular, in the multiple regression model, *UCP1*-VAT alone explains about 10% of the variation in REE.

**Table 3:** Correlation and regression analyses of Resting Energy Expenditure (REE) in 35 patients with obesity.

	**REE (kcal/day)**	
	**Simple linear coefficients (*r*)**	**Multiple regression coefficients (β)**
Sex	−0.283	0.255
WC	0.406^*^	−0.0365
FFM	0.45^**^	0.883^***^
FM	0.181	0.321
HOMA	0.472^**^	0.344^*^
HDL	−0.244	0.375^*^
IL-6	0.160	0.315^*^
*UCP1*-VAT	0.1	−0.373^*^

*WC, waist circumference; FFM, Free Fat Mass (kg); FM, Fat mass (kg); HOMA, Homeostasis model assessment-insulin resistance index; HDL, High Density Lipoprotein –cholesterol; IL-6, interleukin-6; UCP1-VAT, uncoupling protein 1 expression in visceral adipose tissue. Simple linear correlations were calculated by Pearson's correlation. We applied to this subgroup the same multiple regression model computed for the main population and introduced in the analysis UCP1-VAT as a possible biological implicating factor. FFM, HOMA, IL-6, HDL, and UCP1-VAT were independent factors correlated with REE (r = 0.736, AdjRsqr = 0.612)*.

* p < 0.05;

** p < 0.01;

*** *p < 0.001*.

## Discussion

Factors influencing REE in humans are still under investigation, particularly for the mechanisms underlying the association with IR. In Pima Indians basal and 24-h energy expenditure (24hEE) adjusted for body composition, spontaneous physical activity, sex and age are higher in individuals with T2DM compared with non-diabetic subjects (6). In the same way, a study including PWO (mean BMI 32 kg/m^2^) from Sudan proved that REE increased in the presence of T2DM (5). These recent findings are consistent with what was previously reported by Weyer et al. regarding the direct role of insulin in thermogenesis (17).

The increment of REE described in subjects with high IR level results in lower rates of weight gain (18) due to the increase in fat oxidation rate (19) and in gluconeogenesis induced by high free fatty acids concentration in blood (6). Furthermore, Piaggi et al. (19) described a positive association between FPG, as well as a marker of impaired glucose tolerance, and REE. In support of these mechanisms, the improvement of glycaemic control causes a significant reduction in REE (20).

In this context, our study aims to analyze the association of REE with clinical and laboratory parameters in a large population of Caucasian patients with severe obesity and to quantify *UCP1* expression as a “brite” marker in abdominal adipose tissue depots to identify possible determinants of energy expenditure.

We firstly considered all variables correlated with REE in 209 PWO and then we performed a multiple regression model, excluding some interrelated variables. Our results confirm that FFM, which reflects the metabolically active tissue, is the major determinant of REE (1, 21). However, FM and HOMA were also found to be independent predictors of REE. In addition, we found that in our PWO, HOMA is associated with REE both in a simple correlation and in a multiple regression analysis. This result has been supported by the further simple correlations between REE and FPG and insulin, that we showed in the same group. These findings confirm the correlation between REE and HOMA highlighted in other studies (3, 22) but our study innovatively enrolled PWO. When we divided our patients according glycaemic profile, we did not find a significant difference in REE between these subgroups. The lack of difference could be accounted for a lower REE in some patients with well-controlled T2DM (20) and the concomitant presence of several patients classified as prediabetic with high fasting glycaemia incrementing their REE values (19).

In our study, we also observed a simple correlation between REE and WC. In agreement with literature, WC can be considered as a measure of VAT (23), a fat depot that seems to play a more significant pathogenic role than SAT in the development of metabolic complications. This result is consistent with a previous work, including only women with obesity, showing a relationship between REE and visceral fat accumulation measured by abdominal computed tomography (24). Accordingly, we found a simple inverse correlation between REE and HDL-cholesterol and we recognized HDL as an independent element influencing REE. Our findings underline the strength of the HDL levels, which are properly used in the definition of metabolic syndrome (25), as a clinical marker of IR associated with abdominal adiposity, predictive of cardiovascular diseases and metabolic complications.

It was reported that inflammation was related to REE (26, 27), probably for the energy costs due to the inflammatory status. We considered the blood levels of IL-6 and hs-CRP as inflammatory markers implicated in obesity and metabolic disorders (28). We found that both factors correlated with REE and we introduced IL-6 levels in our regression model. In fact, new insights supported that IL-6 not only acts as a central mediator of inflammation but also serves as an endocrine modulator of metabolism for the entire body (29). In mice, IL-6 secreted by brown adipocytes was required for effects of BAT on glucose homeostasis (30). Furthermore, very recent findings showed that human beige adipocytes secrete IL-6 to sustain their own differentiation (31). In PWO increased levels of UCP1 and IL-6 expression in BAT were associated with metabolic improvements (32). Interestingly when we introduce *UCP1*-VAT in the regression model, IL-6 appears as a further independent variable correlated with REE.

In a group of 62 patients we studied the expression of *UCP1*, beige-related genes (*TBX1* and *SLC27A1*) and mitochondrial biogenesis markers (*PPARGC1A* and *TFAM*), in SAT and VAT depots collected from the same patients during bariatric surgery and thus we explored a possible role of browning in human WAT as a determinant of REE. We found that *UCP1* mRNA is higher in VAT than in SAT of unstimulated PWO and that it correlates with BMI, being significantly increased in patients with BMI > 50 kg/m^2^. In our analysis, the positive correlation between BMI and *UCP1*-VAT could be explained by the high caloric intake of the patients. These results are supported by studies in animal models, where an increase of UCP1 mRNA and protein during the high fat diet was described, mainly in BAT (33). Moreover, a high fat diet increases the expression of UCP1 together with other “brite” markers in WAT of rats (34). Both phenomena could evidence an adaptation that tries to contrast the increase in adiposity. Nevertheless, data regarding browning in both mice and humans are still controversial and, mainly in humans, under investigation. In fact, in humans, browning was observed in SAT of burn patients and during cancer cachexia, two conditions characterized by hypermetabolism (35, 36). Furthermore, we know that in lean subjects, with a fluoro-deoxyglucose position emission tomography/computerized tomography analysis, BAT was inversely related to BMI and total and visceral fat areas (37–39).

We confirmed in PWO that *UCP1*-VAT was higher than *UCP1*-SAT as reported previously in humans (13, 14). Moreover, we found also an upregulation of beige-related genes (40) (*TBX1* and *SLC27A1*) in VAT, which confirmed that VAT in humans displayed a brite signature in association with increased mitochondrial biogenesis markers (*PPARGC1A* and *TFAM*) (41). Jorge et al. (32) demonstrated that PWO expressed higher *UCP1* in BAT compared to SAT and they evidenced a simple correlation between *UCP1*-BAT and oxygen consumption (VO2). Our results seem to indicate that the expression level of *UCP1* in VAT could be intermediate between SAT (very low) and BAT (very high) in PWO. Moreover, using a multivariate analysis we were able to show that *UCP1*-VAT resulted as an independent determinant of REE, explaining about 10% of the variance in REE. Furthermore, in this model, FM lost its association with REE, suggesting that the contribution of FM to REE could be mediated at least in part by *UCP1*-VAT. The biological role of UCP1 to predict “brite” proprieties of VAT was corroborated by the strong correlations between *UCP1* and *PPARGC1A, TFAM, TBX1*, and *SLC27A1* expression in VAT.

Our findings suggest that, despite mitochondrial degeneration described in IR state (42), *UCP1* expression in VAT could contribute to energy expenditure and counteract further weight gain in patients with severe obesity.

Our study has some limitations. In particular, we did not assess other possible causes of variation of REE, like heritability (2), elevated protein metabolism, activated substrate cycle (4, 6, 17), changes in glucagon levels (43) and central nervous system involvement (44). However, excluding genetic variation, all these proposed explanations can potentially be caused by an IR state (6, 17). Secondly, we estimated the FM of PWO but we did not measure the specific amount of VAT and SAT with imaging techniques. Moreover, we considered *UCP1* expression in WAT as a well-accepted marker of “brite/beige” adipocytes even though recently other mechanisms driving UCP1-indipendent thermogenesis have been described in WAT of mice and humans (45–49) and it would be interesting to evaluate whether they can also contribute to REE.

In conclusion, we confirmed that REE correlates with FFM, FM and HOMA in a large cohort of patients with severe obesity. Furthermore, we evidenced new independent associations between REE and HDL-cholesterol, IL-6, and *UCP1*-VAT. Moreover, we showed that VAT of patients with obesity expresses more *UCP1* than SAT, incrementing with increase of BMI (BMI > 50 kg/m^2^). Thus, we could hypothesize that abdominal omental VAT in humans is more prone to diet-induced browning. Lastly, we suggest that an increased REE and browning in metabolically complicated severe obesity could represent an effort to counteract further weight gain. Further studies in a larger population analyzing *UCP-1* expression upon stimulation could be necessary to confirm and extend our preliminary results.

## Materials and Methods

### Patients

The study design was schematically represented in Figure 1.

Two hundred and nine Caucasian PWO were enrolled at the Center for the Study and Integrated Treatment of Obesity, Padua University Hospital in the period 2013–2017, with a BMI >30 kg/m^2^. Patients underwent a multi-disciplinary evaluation according to a standard clinical protocol and a complete medical history was taken regarding eating, physical activity, smoking and drinking habits, drugs and medications, past and current medical conditions. PWO with a BMI >35 kg/m^2^ in the presence of co-morbidities or with a BMI >40 kg/m^2^ were candidates for bariatric surgery according to European criteria (50).

Specific exclusion criteria for this study were diagnosis of cancer in the previous 5 years, thyroid hormones imbalance, presence of infections or chronic inflammatory diseases, abuse of caffeine (more than three coffees/day), use of weight-loss drugs and other drugs that could interact with REE.

All subjects gave written informed consent in accordance with the Declaration of Helsinki. The protocol was approved by the “Padua Ethical Committee for Clinical Research” (2892P, 10/06/2013).

### Anthropometric Measurements

All anthropometric measurements were taken with subjects wearing only light clothes without shoes. Height was measured to the nearest 0.01 m using a stadiometer. Body weight was determined to the nearest 0.1 kg using a calibrated balance beam scale. Waist circumference was assessed using a tape measure and BMI was calculated as weight (kg) divided by height-squared (m^2^).

### Energy Expenditure

REE was measured with indirect calorimetry in 209 PWO (described in Table 1) in fasting condition and after 15 min of rest in a comfortable and thermo-neutral environment. Rigorous attention to the standardization of measurement conditions was given, including ensuring that subjects avoided exercise, stressful situations or stimulants at defined intervals prior to the test (51, 52). A ventilated canopy calorimeter was used (Vmax – Sensormedics, Milan, Italy). The system was calibrated before every measurement according to the instructions provided by the supplier. Oxygen uptake and carbon dioxide production were measured continuously and values were averaged at 1-min intervals. REE and respiratory quotient were calculated by using the Weir equation (53).

### Body Composition

Body composition was analyzed with Body Impedance Assessment (BIA) by using a single frequency (300 μA, 50 kHz) electrical impedance analyzer (Soft Tissue Analyzer, Akern, Pontassieve, Italy) in the same conditions as indirect calorimetry. Resistance (R), reactance (Xc) and the phase angle were registered and fat-free mass (FFM) and fat mass (FM) were derived using the software provided by the manufacturer (Bodygram software, Akern, Pontassieve, Italy).

### Biochemical Measurements

For each patient we measured FPG, insulin, lipid profile [total cholesterol (TC), HDL- and Low Density Lipoprotein-cholesterol (LDL-cholesterol), triglycerides (TG)], hs-CRP, IL-6, Tumor Necrosis Factor-α (TNF-α), and Leptin. All blood tests were performed after 8-h fasting. Venous blood samples were collected in tubes coated with 68 IU lithium heparin, with EDTA 8% or with acrylic gel/micronized silica in the morning (Becton Dickison, East Rutherford, NJ, USA). Samples were stored at −20°C until analysis.

All biochemical blood analysis has been performed with standard diagnostic kit according to the WHO First International Reference Standard: glucose (Glucose HK Gen.3, Roche Diagnostic, USA), insulin, IL-6, TNF-α (IMMULITE 2000 Immunoassay, Siemens Healthcare GmbH, Germany), hs-CRP (CardioPhase High Sensitivity C-Reactive Protein, Siemens Healthcare), and Leptin (Leptin-RIA-CT, Mediagnost, Germany). Serum lipids were measured by spectrophotometer (Roche Diagnostic, USA). LDL cholesterol was calculated according to Friedewald (54). Non-HDL-cholesterol (CNHDL) has been obtained by the difference between TC and HDL-cholesterol. A 3-h 75 g oral glucose tolerance test was performed monitoring blood glucose, insulin plasma levels at basal time and 30, 90, 120, 150, and 180 min after glucose load (180 ml of syrup with 82.5 g glucose monohydrate equal to 75 g of glucose) according to WHO standards (15).

HOMA was used to calculate the IR index by: [fasting serum insulin (μU/ml) × fasting plasma glucose (mmol/l)]/22.5, as previous described (55). In patients affected by T2DM, if insulin treated, fasting insulin was not measured and HOMA was not calculated.

### Human Adipose Tissue Samples

During bariatric surgery paired SAT and VAT biopsies were collected in 62 PWO (described in Table 1), immediately frozen in liquid nitrogen and stored at −80°C until RNA extraction. AT surrounding pheochromocytoma was collected during surgery for neoplasm removal.

### RNA Extraction and Reverse Transcription

Total RNA was extracted using the RNeasy Lipid Tissue Mini Kit (QIAGEN GmbH, Hilden, Germany) following the supplier's instructions and quantified using NanoDrop (Thermo Fisher Scientific, Waltham, MA, USA).

One μg of RNA was treated with DNase Treatment & Removal Reagents (Thermo Fisher Scientific) and reverse transcribed for 1 h at 37°C in a 50 μl reaction containing 1X RT buffer, 150 ng random hexamers, 0.5 mM dNTPs, 20 U RNAsin Ribonuclease Inhibitor, and 200 U Moloney Murine Leukemia Virus reverse transcriptase (all from Promega Corp., Madison, WI, USA).

### qPCR

PCR was carried out with Platinum SYBR Green qPCR SuperMix-UDG (Thermo Fisher Scientific) on DNA Engine OpticonTM2 Continuous Fluorescence Detection System (MJ Research, MA, USA). Reaction conditions and primer sequences were reported in Table 4. Each sample (5 ng of cDNA) was assayed in duplicate and quantified using a standard curve method. Results were normalized to HMBS mRNA content and reported as arbitrary unit ratio (target/housekeeping). The melting curve analysis and a positive control (*UCP1*: VAT surrounding pheochromocytoma, *PPARGC1A* and *TFAM*: liver, *TBX1*: muscle, *SLC27A1*: white adipose tissue) were always included to check PCR specificity.

**Table 4:** qPCR conditions.

**Official symbol**	**Forward (5^**′**^-3^**′**^)**	**Reverse (5^**′**^-3^**′**^)**	**Primer (F/R nM)**	**Condition**	**Amplicon (bp)**
*UCP1*	CTA CGA CAC GGT CCA GGA GT	GCC CAA TGA ATA CTG CCA CT	300/300	95°C × 15″ 60°C × 1′ 40 cycles	110
*HMBS*	GGC AAT GCG GCT GCA A	GGG TAC CCA CGC GAA TCA C	300/300	95°C × 15″ 60°C × 1′ 40 cycles	60
*PPARGC1A*	CAG CCT CTT TGC CCA GAT CTT	TCA CTG CAC CAC TTG AGT CCA C	300/300	95°C × 15″ 60°C × 1′ 40 cycles	101
*TFAM*	AGC TCA GAA CCC AGA TGCA A	TTT ATA TAC CTG CCA CTC CGC C	300/300	95°C × 15″ 60°C × 1′ 40 cycles	127
*TBX1*	ACG ACA ACG GCC ACA TTA TTC	TGA ATC GTG TCT CCT CGA ACA	300/300	95°C × 15″ 60°C × 1′ 40 cycles	141
*SLC27A1*	TAC CAC TCG GCA GGA AAC ATC ATC	TGA ACC ACC GTG CAG TTG TAC TT	300/300	95°C × 15″ 63°C × 1′ 40 cycles	131

*Primer sequences and concentrations, reaction conditions and amplicon sizes are outlined for each mRNA quantified. UCP1, uncoupling protein 1; HMBS, hydroxymethylbilane synthase; PPARGC1A, PPARG coactivator 1 alpha; TFAM, transcription factor A, mitochondrial; TBX1, T-box transcription factor 1; SLC27A1, solute carrier family 27 member 1*.

### Statistical Analysis

Statistical analyses were performed using the Systat Software SigmaPlot v.13. Data are presented as mean values ± standard deviations or as median values (25th−75th percentile). All variables were tested by normal Test (Shapiro-Wilk test) and Equal Variance Test (Brown-Forsythe). Pearson's correlation coefficient (r) and the relative *p*-values were calculated to analyze simple linear correlations between two variables. A multiple regression model was computed with REE as the dependent variable and the variables found to be simple correlated with REE as independent variables. This model was used to analyze a subgroup of 35 PWO in which we quantified also *UCP1* mRNA in VAT biopsies (*UCP1*-VAT) and entered this new variable as an independent variable. Differences between the population of 209 patients vs. the subgroup of 35 patients were analyzed by Mann-Whitney *U*-test for independent samples in non-normally distributed variables, independent *t*-test in normally distributed variables and Fisher's exact test in categorical variables. Differences between *UCP1* expression in VAT and SAT were analyzed by the Mann-Whitney *U*-test. In all analyses, the *p*-values were two-sided and a *p*-value lower than 0.05 was considered statistically significant.

## Data Availability

The datasets generated for this study are available on request to the corresponding author.

## Ethics Statement

All subjects gave written informed consent in accordance with the Declaration of Helsinki. The protocol was approved by the Padua Ethical Committee for Clinical Research (2892P, 10/06/2013).

## Author Contributions

SB and FF conceptualized the study, the methodology, and prepared the original draft. SB, FF, LB, AB, and MS participated in the data curation and the statistical analyses. CC, RF, RS, CD, LP, and MF provided the clinical data and samples. RV, GM, and LB supervised the study, reviewed, and edited the manuscript.

### Conflict of Interest Statement

The authors declare that the research was conducted in the absence of any commercial or financial relationships that could be construed as a potential conflict of interest.

## Abbreviations

AT: adipose tissue
BAT: brown adipose tissue
BIA: Body Impedance Assessment
BMI: Body Mass Index
CNHDL: non-HDL-cholesterol
F: female
FFM: Free Fat Mass
FM: Fat mass
FPG: Fasting plasma glucose
HDL: High Density Lipoprotein –cholesterol
HMBS: hydroxymethylbilane synthase
HOMA: Homeostasis model assessment-insulin resistance index
hsCRP: high-sensitivity C-Reactive Protein
IL-6: interleukin-6
IR: insulin resistance
LDL: Low Density Lipoprotein –cholesterol
M: male
OGTT: oral glucose tolerance test
PPARGC1A: PPARG coactivator 1 alpha
PWO: patients with obesity
REE: resting energy expenditure
RQ: respiratory quotient
SAT: subcutaneous adipose tissue
SLC27A1: solute carrier family 27 member 1
T2DM: Type 2 diabetes mellitus
TBX1: T-box transcription factor 1
TC: total cholesterol
TFAM, transcription factor A: mitochondrial
TG: triglycerides
TNF-α: Tumor Necrosis Factor-α
UCP1: uncoupling protein 1
VAT: visceral adipose tissue
VO2: Oxygen Consumption
WAT: white adipose tissue
WC: waist circumference.
